# A Randomized Sham-Controlled Mixed Methods Pilot Study of the Feasibility of Acupuncture for Chemotherapy-Induced Neuropathy: Lessons Learned From Patient Experiences in Integrative Cancer Care

**DOI:** 10.1177/15347354231178877

**Published:** 2023-06-09

**Authors:** Kristina Lagerstedt, Anna Efverman

**Affiliations:** 1 Region of Kronoberg, Växjö, Sweden; 2 University of Gävle, Gävle, Sweden

**Keywords:** acupuncture, pain, neurotoxicity, neuropathy, complementary and alternative therapies, integrative oncology, nursing, physiotherapy, cancer rehabilitation

## Abstract

**Objective:**

Since there is a lack of effective pharmacological therapies for chemotherapy-induced neuropathy and many patients ask for integrative cancer therapies such as acupuncture, the objective of this pilot study was to describe patients’ experiences, and to study the feasibility and short-term effects of genuine acupuncture for chemotherapy-induced neuropathic pain and unpleasant sensations compared to sham acupuncture.

**Methods:**

The pilot study used mixed methods, collecting quantitative and qualitative data. Patients (n = 12) with chemotherapy-induced neuropathy after colorectal cancer were blindly randomized to genuine acupuncture or telescopic sham acupuncture. Individual interviews were conducted, and were analyzed using qualitative content analysis. The patients registered pain and unpleasant sensations (100 mm Visual Analog Scales) before and after n = 120 sessions, n = 60 genuine and n = 60 sham acupuncture sessions.

**Results:**

Five categories of patient experiences were described. The neuropathy negatively affected life. Physical activity was perceived to be important for health, but neuropathy was a barrier. The neuropathy required symptom-managing strategies. Acupuncture was pleasant and valuable, but some patients presented doubts regarding its effect mechanisms. After the genuine acupuncture sessions, pain (mean −2.0 steps relief during each session) and unpleasant sensations (−2.4) in the face was reduced more than after sham acupuncture (+0.1 steps worse pain, *P* = .018, +0.1 steps worse unpleasant sensations, *P* = .036). After genuine acupuncture, unpleasant sensations in the hands were reduced less (−0.23) compared to after sham acupuncture (−5.5, *P* = .002). Pain or unpleasant sensations in the feet did not change.

**Conclusions:**

Patients experienced that the neuropathy negatively changed their life and that acupuncture was pleasant and valuable. Patients receiving genuine acupuncture had short-term effects regarding pain and unpleasant sensations in the face compared to patients receiving sham acupuncture, while hands and feet did not improve. The patients were successfully blinded and complied with the acupuncture. We welcome future full-scaled randomized sham-controlled acupuncture studies.

## Introduction

More than one-third of people in developed countries will be diagnosed with a cancer illness during their lives, and adjuvant chemotherapy after cancer tumor surgery is commonly applied to increase survival. Chemotherapy-induced neuropathy is a painful, unpleasant side-effect of the neurotoxic chemotherapy agents delivered, for example, to patients with colorectal cancer.^1^ Patients with cancer ask for integrative cancer therapies,^2^ such as acupuncture, for many side-effects induced by cancer therapy, including for pain.^3^ However, there is a lack of sham-controlled studies regarding whether acupuncture is feasible for chemotherapy-induced neuropathic pain and unpleasant sensations.

Approximately 60% to 80% of patients undergoing neurotoxic chemotherapy for colorectal cancer experience neuropathy.^4-6^ By adding neurotoxic oxaliplatin, survival increased by approximately 25%, but neuropathy became a frequent and bothering side-effect.^1^ Oxaliplatin is suggested to be absorbed in dorsal root ganglia resulting in a sensory neuropathy, inducing a disturbed microcirculation and inflammatory processes in the peripheral nerve endings.^7^ The neuropathic symptoms occur during or after the chemotherapy and consist of, for example, decreased sensorial functioning, pain, numbness, tingling and other unpleasant sensations in the hands, feet and face, especially around the mouth. The patients often experience cold sensitivity, hyperalgesia, and muscle cramps in these areas.^1,4-6^

Integrative oncology is a patient-centered, evidence-informed field of cancer care that utilizes complementary and alternative therapies from different traditions, such as acupuncture therapy, alongside conventional cancer treatments.^2^ Inadequate response to drug treatments constitutes a substantial unmet need for symptom management in patients with neuropathy.^1,8^ Although a large number of interventions have been studied, there is currently no effective pharmacological therapy,^1,8^ or integrative cancer therapy available for chemotherapy-induced neuropathy, except for physical activity.^8^ Oncologists often have to lower the dose and frequency of the neurotoxic chemotherapy,^1^ since the dose of the neurotoxic agent is the only factor known to affect neuropathy.^9^ This in turn risks to lower the rates of survival.^1^ Most of the anticonvulsants and tricyclic antidepressants, that typically are prescribed for neuropathic pain are not effective for chemotherapy-induced neuropathy.^1,8^ A study (n = 231) demonstrated the effect of the pharmacological agent duloxetine.^10^ However, duloxetine has not been implemented into routine care.^1,8^ According to quantitative data, neuropathy worsens experienced health and quality of life.^4-6,11,12^ The severe symptom burden and the lack of effective medications highlights the need for integrative therapies added to conventional therapies.^2,3,8^ Acupuncture is often requested^3^ and is available for large proportions of patients with cancer, being a part of integrative cancer therapy.^2,8,13^ Acupuncture has been highlighted in randomized but not sham-controlled studies to be a feasibly conducted integrative therapy for chemotherapy-induced neuropathy.^14-17^ Neuropathic pain after chemotherapy decreased and quality of life improved more in patients receiving methylcobalamin injections combined with acupuncture (n = 49) compared to patients receiving methylcobalamin injections but no acupuncture (n = 49).^14^ Neurophysiological, physical, and neuropathy relief improved during acupuncture therapy (n = 30) compared to waiting list controls (n = 30).^17^ Further, pain and symptom burden decreased, and neurological status outcomes and quality of life improved during acupuncture therapy (n = 44) compared to waiting list controls (n = 43).^16^ Improved physical and functional aspects of QoL after receiving acupuncture were also reported.^15^ These studes did not control for non-specific effects surrounding the acupuncture therapy.^18-21^ However, patients receiving genuine electro-acupuncture during their chemotherapy-period reported worse pain-levels than patients receiving sham electro-acupuncture at a 4 month follow-up (n = 48).^22^

The psychoneuroimmunological mechanisms of actions during acupuncture for neuropathy are not yet well understood.^23^ Acupuncture has been hypothetized to reduce neuropathy, potentially by increasing blood flow in peripheral nerve endings, production of endorphins and nerve growth factor, and through immunological effects.^23^ Needle penetration increases the blood flow in the tissue close to the needles.^24^ Acupuncture plausibly stimulates receptors or causes the regular discharge of nerve fibers, leading to peripheral and central nervous system activation, resulting in the release of a variety of neurotransmitters.^14^ Acupuncture is accordingly based on sensorial stimulation, which induces wellbeing effects by social learning mechanisms.^18^ Randomized controlled acupuncture studies are thus warranted to distinguish between effects of the specific ingredients of the acupuncture, that is, skin-penetration at traditional acupuncture points and needle stimulation resulting in the specific needle-sensation,^19-21^ and the social learned wellbeing effects and contextual effects of the entire treatment procedure.^18^ The pathology of sensory neuropathy often involves non-nociceptive sensorial stimuli becoming painful,^25^ which hypothetically explains the increased level of neuropathic pain previously seen during electro-acupuncture.^22^ Thus, preceding future full-scaled sham-controlled studies, it seems important to reach a deeper understanding on the patients’ experiences when receiving acupuncture for chemotherapy-induced neuropathy, and to describe the feasibility and short-term effects of manual acupuncture for chemotherapy-induced neuropathy compared to sham acupuncture. Cancer care practitioners are increasingly practicing acupuncture or informing patients regarding potential effects of acupuncture and need to be aware of patients’ experiences of acupuncture therapy.^26^ Integrative cancer care researchers in general are increasingly using designs combining qualitative and quantitative methods, often called mixed methods research, achieving a “whole greater than the sum of the parts.”^27^

Preceding a full-scaled randomized controlled study, the objective of this pilot study was to study the feasibility of a sham-controlled acupuncture study design. The different aspects of the objective were to: 1. Describe patient experiences of neuropathy and acupuncture treatment for neuropathy; 2. Study the patients’ compliance to the study procedure, the blinding to genuine or sham acupuncture, and potential side-effects or complications, and; 3. Evaluate short-term effects of genuine acupuncture on chemotherapy-induced neuropathic pain and unpleasant sensations compared to sham acupuncture.

## Methods

### Design

This pilot study^28^ used a randomized sham-controlled design, applying mixed methods^27^; both quantitative and qualitative data were collected. In line with recommendations for pilot studies,^28^ we primarily studied several aspects of the feasibility of the study logistics, measures, and intervention/control procedure (i.e., the patients’ compliance to the study procedure and to the blinding to genuine or sham acupuncture, and potential side-effects or complications), and the patients’ experiences. Secondarily to these feasibility aspects, we studied a scientific aspect,^28^ covering the evaluation of short-term effects of genuine acupuncture on the chemotherapy-induced neuropathic pain and unpleasant sensations.

The study complied with the declaration of Helsinki regarding ethical principles for medical research involving human subjects. The feasibility testing of the acupuncture therapy was approved by the regional ethics committee (Linköping, approval number 2014/80-31). The qualitative interview questions focusing on other than acupuncture therapy aspects of the patients’ experiences were ethically reviewed being part of an academic master thesis, in line with the Swedish Statute (2003:615) concerning the Ethical Review of Research Involving Humans.

### Inclusion

In this pilot study, 12 patients participated; pilot studies usually study 10 to 15 patients.^28^ A study-coordinating oncology nurse during 8-month period screened patients who previously had received adjuvant chemotherapy at one Swedish region hospital. Inclusion criteria were individuals of at least 18 years age, who after colorectal cancer surgery had received adjuvant oxaliplatin-based adjuvant chemotherapy 3 to 36 months preceding the inclusion, with persistent chemotherapy-induced neuropathy, that is, occurrence of at least one symptom on the Swedish version^29^ of the Oxaliplatin Neurotoxic Specific Scale,^30^ and with linguistic, mental, and physical capacity to give informed consent. Exclusion criteria were any kind of other neuropathy (e.g., diabetes neuropathy), or a new or recurrence cancer disease at the time for the study, irrespective of type or stage of cancer. Thus, all participants were currently cancer-free. The study-coordinating oncology nurse screened 38 patients: eight did not conform to study criteria, while 19 were given written and oral study information and asked for participation. Seven patients did not want to participate, while n = 12 gave informed consent and participated.

### Randomization and Study Information

A statistician randomized the 12 patients to genuine or sham acupuncture, using a computerized random table. A therapist treated the patients (being outpatients) at the oncology department of the regional hospital 2 times a week for 5 weeks, which is a commonly applied frequency of acupuncture treatment in clinical practice.^31^ This resulted in 10 observed acupuncture treatment sessions per patient, or in total 120 observed sessions; 60 genuine and 60 sham acupuncture sessions. The patients were informed orally and in writing that the beneficial effect of either treatment was not known and that: “You will receive—without being told which—an ordinary acupuncture treatment with needles penetrating the skin or another treatment with needles placed just against the skin.” None of the randomization alternatives were cited as a “sham” or “placebo” treatment. The patients, the evaluator, and all health-care practitioners at the oncology department other than the acupuncture-providing therapist were blinded to the treatment allocation.

### The Genuine and the Sham Acupuncture

The therapist delivered Western medical manual genuine acupuncture bilaterally to traditional acupuncture points located in the face (Stomach 6; ST6), hands (Large intestine 4; LI4, Small Intestine 3; SI3) and feet (Bladder 60; BL60, Kidney 3; KI3), in concordance with previous studies and knowledge regarding areas affected by neuropathy.^1,14-17,20^ In line with Western medical acupuncture, it seemed important to consider proposed neurophysiological effect mechanisms when selecting these acupuncture points.^31^ Sharp needles in stainless steel (manufactured by DongBang, Korea), diameter 0.25 × length 40 mm, were inserted to a depth of a third to a half body-inch, depending on the location of the acupuncture points. One body-inch (one “cun”) is an acupuncture-specific dimension equivalent to the greatest width of the individual patients’ thumb, *approximately* 1.5 cm). The therapist manipulated the needles 3 times (at the start, middle and end of every session) by twirling and lifting until “deqi” occurred. “Deqi” means the specific needle sensation, which was documented in the standardized treatment protocol when the patient reported a sense of numbness or soreness, and the therapist noted a minimal muscular contraction around the needle.^32^

The therapist delivered sham acupuncture^33^ bilaterally to non-acupuncture points^20^ located in the face (2 body-inch proximal to ST6), hands (2 body-inch ulnar to LI4, and 2 body-inch proximal and one body-inch radial to SI3) and feet (the middle point between BL60 and KI3, and 2 body-inch proximal to KI3). The credible^19^ non-penetrating telescopic sham needle “Park et al ’s sham device”^33^ in stainless steel, 0.25 × 40 mm fully extended length (manufactured by DongBang, Korea) was used. The sham needle looks identical to a real needle but is blunted and glides upwards into its handle instead of penetrating, giving an illusion of penetration. “Park’s sham device” has a tube with a bottom plate covered with double-sticky tape marking the needling-points and holding the sham needle in place. The therapist manipulated the sham needles a few seconds 3 times per session resulting in the needles touching the skin, but no “deqi” occurred.^19,32^ Except for placing and manipulating the needle, the sham needle was not pressed against the skin at all, that is, no “acupressure” occurs using the “Park et al ’s sham device.”^33^

The therapist was a physiotherapist (13 years of experience) and had undergone acupuncture education comparable with 10 weeks full-time academic studies. The therapist was trained regarding the standardized sham treatment technique for 2 hours. Preceding this study, the therapist did practice the sham-acupuncture technique during a previous acupuncture study.^34^

### Data Collection

#### Interviews regarding patient experiences

A therapist, who was independent from the acupuncture therapy, performed individual qualitative interviews^35^ guided by a semi-structured interview guide (Table 1) at a follow-up 3 months after the acupuncture therapy. At that time, the patients were still blinded to type of acupuncture. Eleven of the 12 patients participated in this individual interview, while one patient could not attend due to worsened general condition. The interviews were audio-recorded, ranging 25 to 54 minutes (median 37 minutes), and then transcribed verbatim. The interviewer preceding the data collection tested the interview guide during 2 pilot patient interviews (not included in the study). One minor correction was made to clarify the first interview question, that is, “your problems with neuropathy symptoms” was changed to “your neuropathy symptoms,” to not point out that neuropathy must be a problem.

**Table 1.** The Semi-Structured Interview Guide.

Do your neuropathy symptoms affect your life and if so, in what ways?
How do you view your capacity to manage with physical activity in line with recommendations on physical activity?^1^
May you describe your experiences regarding the acupuncture treatment for your neuropathy?

Counter questions were: “Can you tell me more about that?”; “What did you experience?”; “What did you do?.” ^1^Preceding this question, the interviewer said: “Guidelines for persons diagnosed with cancer recommend at least 150 minute of moderate intensity physical activity, or 75 minute of vigorous physical activity, weekly.” After the interview questions, the interviewer summarized the interview and asked: “Did I understand your experiences in an appropriate way, or do you want to add information or to further explain something?.”

#### Descriptive data

We used a valid study-specific questionnaire to collect data on sociodemographic variables (e.g., age, gender), clinical details (cancer therapy data, previous acupuncture experience and treatment expectations), and the health data described below. The study-specific questions were tested for validity during patient interviews (non-published data).

#### Measuring of pain and unpleasant sensations

Immediately before and after every acupuncture treatment session, the patients in writing graded the neuropathy pain intensity in face, feet and hands, using three 100 mm Visual Analog Scales (VAS; 0= “No pain” to 100=“Worst imaginable pain”). Further, levels of unpleasant sensations in face, feet and hands were graded, using 3 100 mm VAS (0=“No discomfort” to 100=“Worst imaginable discomfort”). Previous studies presented satisfactory validity, reliability and feasibility^36^ of VAS for measuring of pain and unpleasant sensations.

#### Measuring of self-rated health

At the start and the end of the 5-week treatment period, the patients in writing graded their self-perceived health status on the Swedish version^37^ of the widely used valid and reliable Euro-Qol health VAS,^38^ ranged from 0 (worse possible health status) to 100 (best possible health status).

#### Measuring of belief in acupuncture effects

Preceding the first acupuncture treatment session, the patients in writing answered an expectancy question^34^: “Do you believe the treatment to be effective for neuropathy symptoms?” (“No, I do not believe the treatment to be effective” or “Yes, I believe a little”/ “moderately”/“Much”/“Entirely” the treatment to be effective”). The expectancy question presented satisfactory test re-test reliability (Spearman’s correlation coefficient between test and re-test was .85) and satisfactory content validity (Kappa coefficients for agreement to other expectancy scales varied .70-.75).^34^

#### Measuring of side-effects and blinding statement

Previously used weekly written questions covered experience of negative side-effects (“Yes” or “No”) close to the acupuncture sessions and the therapist during every acupuncture session documented if the patents experienced any negative side-effects (bleeding around needles, dizziness, fainting, or other needle-induced unpleasant sensations; yes or no). At the end of the treatment period, the patients graded the overall intensity of needle-induced pain (induced by the skin penetration and the needle stimulation): “Not painful” or “Mildly”/“Moderately”/“Very painful”. A blinding statement was collected: “Do you think you have been treated with needles that have penetrated the skin, or do you think the needles have been placed just against the surface of your skin?.” The patients graded how sure they were on their statement using the alternatives “Not sure at all, just guessed,” “Fairly sure,” or “Entirely sure.”

### Data Analyses

We applied mixed methods, including qualitative and quantitative and data analyses.^27^ Descriptive data for the genuine and the sham acupuncture group regarding sociodemographic and clinical variables (number, n, and percentages, %) were presented. We analyzed the qualitative interview data using qualitative content analysis.^39^ The both authors read the transcripts of the qualitative interviews several times to create a sense of the whole and to pick out phrases and sentences with information relevant to the objective, so called meaning units. The second author condensed the meaning units to compress the text without losing its content and coded the condensed meaning units. Then the authors placed the condensed meaning units into groups, representing different categories. First, the analyzing authors made this procedure independently of each other. Then the categories were discussed until agreement occurred regarding that the categories reflected the central message of the content in the transcribed interviews, in the light of the objective. As a validation,^39^ another senior researcher in cancer care read the analysis and confirmed that it was valid in the light of the content of the transcribed interviews. We visualized the experiences using citations, especially those that were particularlycharacteristic for the category, and labeled the citations by the patient’s code number, used to replace his/her name.

We calculated n and percentages of post-treatment VAS grading that represented lower intensity of neuropathic pain and unpleasant sensations in the face, feet and hands, compared to the pre-treatment session grading (6 categorized variables (“Yes”/“No”): “Reduced pain face,” “Reduced discomfort face,” “Reduced pain feet,” “Reduced discomfort feet,” “Reduced pain hands,” “Reduced discomfort hands”). Further, we calculated the size of the changes (mean, m, and Standard Deviation, SD, of change) between pre and post treatment session VAS grading, in line with recommendations on statistics,^40^ especially for pilot studies.^28^ The Mann Whitney U-test, suitable for skewed data,^41^ was used to compare the genuine and sham acupuncture sessions. We descriptively presented number and percentages of the patients who experienced reduced pain and discomfort, and improved self-rated health, respectively, after the entire genuine or sham acupuncture period (10 treatment sessions), without performing any statistical significance testing due to expected lack of statistical power. Further, we calculated the size of improved self-rated health, presented as a median value and Inter Quartile Range (IQR) for the genuine and the sham acupuncture group. The Statistical Package for the Social Sciences for Windows version 23.0 was used. The significance level was set at 5%; *P*-values of ≥ .05 were considered as not statistically significant. The evaluator was blinded to type of acupuncture (genuine or sham) until the group comparisons were conducted, by use of codes representing each randomization group.

## Results

### Patient Characteristics

The descriptive characteristics of the patients receiving genuine acupuncture and sham acupuncture are presented in Table 2. The genuine acupuncture group compared to the sham acupuncture group consisted of a higher absolute number of men and patients consuming medications other than cancer-related medication.

**Table 2.** Characteristics of the Patients Receiving Acupuncture After Adjuvant Chemotherapy for Colorectal Cancer.

Variables	Genuine acupuncture n = 6	Sham acupuncture n = 6
Time since the former chemotherapy in month, md, IQR	4, 3-5	10, 7-12
Debut of neuropathy, n (%)		
During the chemotherapy period	6 (100)	5 (83)
After the chemotherapy period	0 (0)	1^a^(17)
Sex, n (%)		
Man	5 (83)	2 (33)
Women	1 (17)	4 (67)
Age in years: md, IQR range	68, 63-76	69, 64-70
Marital status, n (%)		
Married or living together	5 (83)	4 (67)
Single	1 (17)	2 (33)
Educational level, n (%)		
Elementary school	2 (33)	3 (50)
Secondary school	2 (33)	2 (33)
Higher education/University	2 (33)	1 (17)
Employment Status, n (%)		
Employed, not on sick leave	0 (0)	0 (0)
Employed, but on sick leave	3 (50)	1 (17
Retired	3 (50)	5 (83)
Medication for any illness other than cancer-related, n (%)		
Yes	4 (67)	3 (50)
No	2 (33)	3 (50)
Previous experience of acupuncture, n (%)		
Yes	3 (50)	3 (50)
No	3 (50)	3 (50)

Abbreviations: md, median; n, number; IQR, Inter Quartile Range.

a The patient’s debut of neuropathy was 10 weeks after the chemotherapy.

### Patient Experiences

Six categories described the content of the qualitative interviews, presenting that the patients experienced that the neuropathy has changed their life and acupuncture was valuable although some patients presented doubts regarding its effect mechanisms (Table 3).

**Table 3.** Categories and Citations Describing the Patients’ Individual Experiences.

Category	Citations
The neuropathy negatively affects life	“ *After all, my feet are like clogs. . .//. . . on the underside, I feel like the sole of the foot is made of wood” (Respondent 5)* “*You couldn’t open the fridge and you couldn’t be in cold water” (Respondent 13)* “*It feels very much, almost more sensitive . . . than I was before I got these troubles” (Respondent 2)* “ *It made a huge dent in my hands! Insanely bad!” (Respondent 9).* “*The symptoms are there all the time” (Respondent 4)* “*It looks healthy as usual” (Respondent 13, about the invisible neuropathy).* *“It feels like sand under the feet” (Respondent 9)*
	“*I still cannot hold the sewing needle” (Respondent 9)* *“My foot could not even recognize that it was a pet on the floor” (Respondent 11, talking about a falling accident)* “*I decided to stop driving the car; I found out that I do not master it anymore” (Respondent 12)*
Physical activity is important for health, but neuropathy is a barrier	“ *You should stay active for the sake of your body. . .//. . .both mentally and physically” (Respondent 13).* “*Then I ’ll walk three days a week, for at least a hour, and then I go at a high pace.” (Respondent 7)* “*I have been walking almost every day, until I could start running again” (Respondent 9)* *“I’ll go for a walk 1 hour a day” (Respondent 4)* “*Those days that it is not outdoor weather. . .//. . ., then I will be on the cross trainer instead (Respondent 2)* “ *I have to bite in and walk anyway” (Respondent 3)* “*I walk approximately one hour a day” (Respondent 11)*
	“*I have stopped bicycling. . ., I miss the bicycling now during summer time” (Respondent 12)* “*The desire to do things has disappeared with it” (Respondent 1).* “*I’m lazy” (Respondent 8)*
The neuropathy requires for symptom managing strategies	“*When it was plus ten degrees*^a^, *you already had to wear double mittens inside as well”(Respondent 9)* “*Do I need information or help then I actively seek it; I don’t sit and wait being angry that I would not get it, so to speak”( Respondent 6)* “ *My wife treats them. Massage and donate. It benefits me” (Respondent 4)* *“I do it to get rid of some of the pain” (Respondent 8)*
	“*I have started learning how to live with it” (Respondent 11)* *“I had to quit my job, not because of my cancer but because of this*^b^*” (Respondent 8)*
Acupuncture was pleasant and valuable	“*During the period of acupuncture therapy, I significantly improved in my hands, that’s for sure (Respondent 11)* “*Especially the feet have been better” (Respondent 1)* “*I have to say that I have been satisfactorily taken care of” (Respondent 7)* “*I perceived it to be very sympathetically and pleasant” (Respondent 11)*
Presenting doubts regarding the mechanisms of acupuncture effects	“*If it was the acupuncture or if it was the time that made the effects, I cannot really say” (Respondent 11)* *“I actually think that the acupuncture helped me but it was more like that I had something to do, distracting my pain” (Respondent 3)* *“I have previously been helped by acupuncture, but not this time. . .; maybe it was due to my worse situation. . .” (Respondent 2)* *“I have previously been treated by acupuncture so I think it can help” (Respondent 1)* *“I really wished the acupuncture to help me” (Patient 5; genuine acupuncture)* *“I’ve tried to find benefits of it. . ., but I’m not really sure I’ve gotten any better in my toes” (Respondent 4)* *“I cannot say that it has helped me” (Respondent 5)* *“One should show respect to the old history of the healing acupuncture. . ., it probably help other people in other situations” (Respondent 6)* *“It has not given me anything. . ., I just become stiff in my needled thumbs (Respondent 7)* *“I don’t know if I have been helped by it, or if the symptoms naturally disappear so it’s hard to say” (Respondent 9)*

a The respondent referred to the temperature, measured in degrees of Celsius.

b Referring to the neuropathy symptoms.

#### The neuropathy negatively affects life

The neuropathy was experienced as constantly causing discomfort and pain. The patients described that they could not coordinate their fingers. They had decreased sensibility for tactile input, and it was difficult to recognize the shape and condition of materials in the way they used before chemotherapy. For example, they could not discriminate between wet and dry materials. Some patients described that their feet seem to being made of wood, a “non-human” material. Their hands were not perceived as being part of their own body anymore. The patients described an increased sensitivity for cold, and a general hypersensitivity. They described that they could not trust their own hands; their fingers were “unreliable.” Suddenly, the fingers made involuntary movements, leading to dropping things during daily activities. Activities such as sewing or driving the car were sometimes not possible to perform. Some patients described that they had not been able to go back to work, not because of the former cancer but because of the neuropathy. One patient described a fallingaccident resulting in a bone fracture. The respondent hypothesized that the falling was a consequence of the balance disturbance combined with decreased sensory perception, since the patient did not recognize climbing on a pet on the floor. Other described being stopped by the police; accidently supposed to be drunk while driving, after walking with poor balance to the car. These situations of dropping things or being out of postural control were described as both impairing and embarrassing. The patients mentioned the complicated paradox that although their neuropathy symptoms constantly induced burden to them, the symptoms were still invisible to others, for example their next-of-kin and the surrounding society. The patients described that they had been forced to change their daily life due to the negative impact of the neuropathy. In their former life, they did not have to think of their own functioning in daily life, they could just live. Nowadays, they had to take the neuropathy symptoms and how they negatively affect daily and occupational life into account constantly.

#### Physical activity is important for health, but the neuropathy is a barrier

The patients perceived physical activity to be beneficial for health. They thus frequently tried to practice physical activity to the best that they possibly could manage. Physical activity, for example taking brisk walks, was very important for them. Many patients described that they practiced walking every day, or several times per week. They practiced self-talk to peer themselves to overcome barriers, to be able to stay physically active. The patients preferred to practice outdoor physical activity. The only exception was if the weather was too bad; those days they chose to practice indoor physical activity. The patients were very aware of benefits of physical activity and hypothesized that their physical activity may lead to better health and even longer survival. The neuropathy however impaired physical activities that used to be done before the cancer, for example, bicycling and walking in the forest. Thus, they have had to interrupt such demanding activities. Going for a stroll on cobbled streets or out in the forest was not possible anymore. The patients experienced pain due to the neuropathy symptoms in the feet during physical activity, inclugin walking. Walking was thus hard to practice now and then, especially walking on uneven ground. Others described that they had stopped using the bicycle for transport. The patients claimed that they might be too impaired, lazy, or unmotivated to adhere to the amount and intensity of physical activity described in guidelines regarding physical activity, due to their neuropathy.

#### The neuropathy requires for symptom managing strategies

The patients described that the burden of the neuropathy forced them to practice symptom managing strategies, aimed to reduce the symptom burden in their daily life. Some strategies covered adjusting compared to before the cancer, for example wearing gloves indoors to avoid being cold. Other strategies were actively managing the neuropathy through seeking care and advice. The patients described distracting strategies, for example moving the feet constantly, resulting in distraction of the pain. Other respondents lubricated ointments on their feet or practiced self-massage. Their next of kin were highly involved in their symptom managing strategies.

#### Acupuncture was pleasant and valuable

The patients described that their acupuncture therapy was pleasant. The feeling of being taken care of by the acupuncture-delivering therapist was also experienced to be valuable. Some patients noticed valuable improvements regarding their neuropathy symptoms; they described that pain and unpleasant sensations in the hands and feet have significantly been relieved when undergoing the acupuncture therapy.

#### Presenting doubts regarding the mechanisms of acupuncture effects

The patients expressed that they were not sure if it was the acupuncture that had been effective, or if improvements had occurred due to the natural history. Some patients claimed that they had not noticed effects of their acupuncture therapy but believed that acupuncture would help other patients. Several patients claimed that they believed acupuncture to be effective, since they previously experienced positive effects from acupuncture.

### Short-Term Acupuncture Effects on Neuropathic Pain and Unpleasant Sensations

After the genuine acupuncture sessions, the patients experienced reduced pain (mean −2.0 steps relief each observation) and unpleasant sensations (-2.4) in the face compared to after the sham acupuncture sessions (+0.1 steps worse pain, *P* = .018, +0.1 steps worse discomfort, *P* = .036) (Table 4). After the genuine acupuncture sessions, the reduction of pain in the hands (−0.37) and feet (−2.6), and the reduction of unpleasant sensations in the feet (-3.4) did not differ compared to the level of reduction seen after sham acupuncture (pain hands: −1.7, *P* = .461, pain feet: −3.9, *P* = .431, discomfort feet −5.5, *P* = .189). After genuine acupuncture, unpleasant sensations in the hands were reduced less (−0.23) compared to after sham acupuncture (−5.5, *P* = .002). The frequency of the genuine and the sham acupuncture sessions resulting in reduced level of pain or unpleasant sensations are presented in Table 4.

**Table 4.** Changes in Neuropathic Pain and Unpleasant Sensations Post Compared to Pre Genuine and Sham Acupuncture Sessions.

Variables	Genuine acupuncture n = 60 sessions			Sham acupuncture n = 60 sessions		
	Pre each treatment session mean ±SD	Post each treatment session mean ±SD	Change mean ±SD VAS, Range, Reduction in n (%) of sessions	Pre each treatment session mean ±SD	Post each treatment session mean ±SD	Change mean ±SD VAS, Range, Reduction in n (%) of sessions
Face						
Pain	4.7+7.7	2.7 + 2.7	−2.0 ± 6.2 −37 - 2 24 (44)	1.6 + 2.0	1.7+2.4	0.1 ± 1.8 −6 - 4 15 (26)
Unpleasant sensations; discomfort	5.5 + 10.6	3.1 + 3.6	−2.4±8.9 −47 - 14 23 (43)	1.4+1.8	1.6 + 2.3	0.1 ± 1.2 −4 - 3 12 (21)
Hands						
Pain	17.2 + 17.7	16.4+17.9	−0.4±11.7 −47 - 48 24 (44)	5.1 + 10.1	2.3 + 2.7	−1.7±4.3 −20 - 2 16 (34)
Unpleasant sensations; discomfort	36.6+19.4	37.0 + 21.6	−0.2 ± 6.2 −16 - 13 24 (45)	20.6 + 22.3	16.3 + 22.8	−4.1±6.8 −29 - 6 39 (68)
Feet						
Pain	16.7+19.4	14.2 + 16.1	−2.6 ± 6.8 −32 - 11 26 (50)	14.0 + 19.2	8.7 + 11.8	−5.6 ± 16.2 −100 - 5 31 (53)
Unpleasant sensations; discomfort	51.0 + 16.3	46.5 + 19.1	−3.4±7.1 −36 - 8 32 (62)	49.1+29.4	43.6 + 31.0	−5.5 + 9.9 −49 - 12 36 (63)

Mean, ±SD and range of changes on VAS score between pre and post treatment sessions are presented; - represents reduced pain/unpleasant sensations. Lack of - preceding a number represents worsened pain/unpleasant sensations. Further, n and proportions (%) of sessions resulting in reduced pain or unpleasant sensations are presented.

Abbreviations: n, number; SD, Standard Deviation; VAS, Visual Analog Scale (0 no pain/discomfort to 100 worse imaginable pain/discomfort).

### Changes in Neuropathic Pain and Unpleasant Sensations With Time

**Figure 1.**
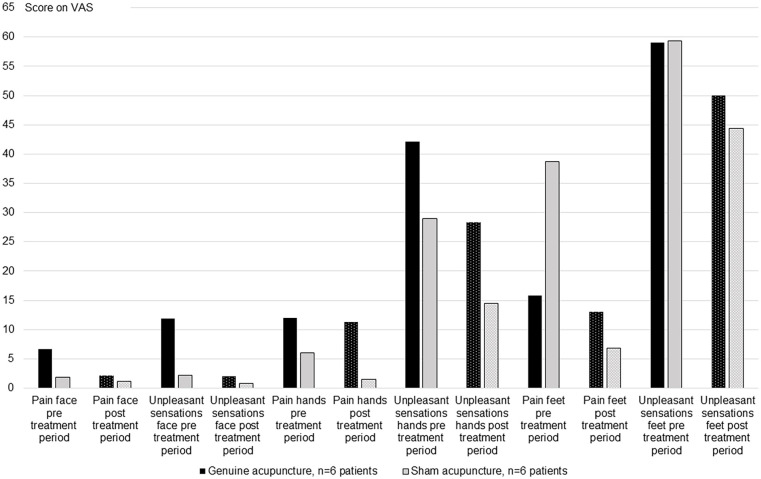
Changes in neuropathic pain and unpleasant sensations post compared to pre the entire treatment period in patients receiving genuine (n = 6) and sham acupuncture (n = 6). Abbreviations: n, number; VAS, Visual Analog Scale: 0 no pain/discomfort to 100 worse possible pain/discomfort.

As descriptively presented in Figure 1, the patients graded the levels of unpleasant sensation to be higher than the levels of neuropathic pain, in the face, hands and feet. Absolute level of graded neuropathic pain and unpleasant sensations decreased with time between the assessment preceding the first genuine or sham acupuncture treatment session and the assessment after the last treatment session.

### Self-Rated Health

**Figure 2.**
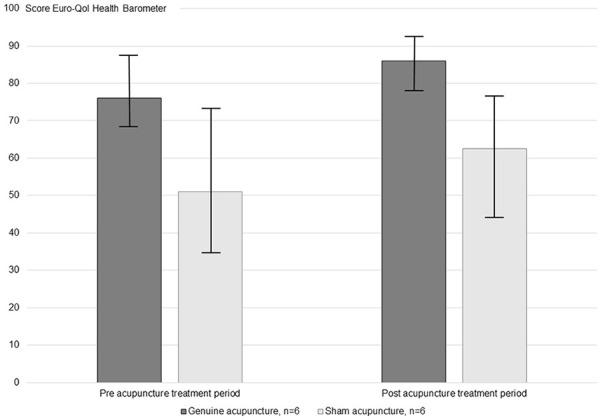
Self-rated Health (Median and Inter Quartile Range) Graded on the Euro-QoL Health Visual Analog Scale (VAS) Pre and Post the Entire Treatment Period in Patients Receiving Genuine (n = 6) and Sham Acupuncture (n = 6). Health VAS: 0 (worse possible health status) to 100 (best possible health status). Abbreviations: n, number; QoL, Quality of Life.

During the treatment period, 4 of 6 patients (67%) in the genuine acupuncture group and 4 of 6 patients (67%) in the sham acupuncture group improved in grading of self-rated health (Figure 2). The genuine acupuncture group improved median 10 steps (IQR 2-11) and the sham acupuncture group improved median 2 steps (IQR 1-19).

### Compliance, Side-Effects, and Blinding Success

All 12 patients complied with the 10 planned acupuncture therapy sessions. Negative side-effects were minor and infrequent. Five of six patients (83%) in both the genuine and the sham acupuncture group considered the needling to be not or just mildly painful. Six (100%) of the genuine acupuncture treated and 5 (83%) of the sham acupuncture treated patients, thought they had received a genuine penetrating acupuncture treatment (Table 5). The sham acupuncture treated patient (n = 1) who guessed that non-penetrating needles were used still strongly believed the acupuncture to be effective.

**Table 5.** Acupuncture Beliefs, Side-Effects, and Blinding Statements.

Needling-related variables	Genuine acupuncture	Sham acupuncture
Treatment expectations; believed acupuncture to be effective for neuropathy pre-treatments	n = 6	n = 6
Believe much	1 (17)	4 (67)
Believe moderately	4 (67)	0 (0)
Believe a little	1 (17)	2 (33)
Do not believe	0 (0)	0 (0)
Negative side-effects, occurring in number (%) of observed patients at least once during the treatment period	n = 6	n = 6
Soreness around at least one needle point	2 (33)	0 (0)
Tiredness	3 (50)	4 (66)
Dizziness	1 (17)	1 (17)
Fainting	0 (0)	0 (0)
Other side-effects	0 (0)	2 (33)^a^
Negative side-effects, occurring in number (%) of observed treatment sessions	n = 60	n = 60
Bleeding around at least one needle point	6 (10)	0 (0)
Tiredness	12 (20)	11 (18)
Dizziness	5 (8)	6 (10)
Fainting	0 (0)	0 (0)
Other; Needle scratched the skin	0 (0)	4 (7)
Overall needle-induced pain^b^		
Not painful	2 (33)	3 (50)
Mildly painful	3 (50)	2 (33)
Moderately painful	1 (17)	1 (17)
Very painful	0 (0)	0 (0)
Blinding statement, n (%)	n = 6	n = 6
Penetrating the skin	6 (100)	5 (83)
Placed against the skin	0 (0)	1 (17)^c^

N of patients answering the questions and n of observations (6 patients in each group a’ 10 treatments = 60 observations) are presented.

Abbreviations: n, number.

a The experienced side-effects were hot flushes (n = 1) and feeling emotional (n = 1).

b Overall pain induced by placing and stimulating the needles.

c This patient stated “I am not sure at all, I guess,” regarding certainty in the statement.

## Discussion

This pilot study described that the patients receiving acupuncture for chemotherapy-induced neuropathy experienced that the neuropathy negatively changed their life and that acupuncture was pleasant and valuable, but some patients presented doubts regarding its effect mechanisms. Patients receiving genuine acupuncture experienced short-term effects regarding pain and unpleasant sensations in the face compared to patients receiving sham acupuncture, while hands and feet did not improve. The patients were successfully blinded and complied with the acupuncture. Since this pilot study just studied patient experiences, feasibility aspects and short-term effects, we do not know if acupuncture in the long-term would be more effective than sham acupuncture for chemotherapy-induced neuropathy. The generalizability of our findings is limited.

The patients during the interviews expressed that the neuropathy negatively changed their life. Some of our patients described that they had been forced to make dramatic life changes due to the neuropathy, for example that they had stopped working, and were not able to bicycle or walk on uneven ground, for example out in the nature anymore. A literature review found that several individual, work-related, and healthcare/society system factors were associated with prolonged sick leave and disability pension post cancer, cancer-related symptoms amongst other aspects.^42^ An event of falling, leading to a bone fracture, was described. Patients with chemotherapy-induced neuropathy have been seen to be overrepresented in accidents.^5^ In persons (n = 512) who had persistent chemotherapy-induced neuropathy 6 years after chemotherapy, increasing symptom severity was linearly associated with worsening function, and higher fall risk.^43^ These impairments have also been confirmed by another study.^44^ Previous studies noticed that patients perceive to not have been sufficiently warned of the risk of untreatable neuropathy induced by the oxaliplatin-based chemotherapy, which saves lives but changes the patient’s life in so many ways.^45^ The observation that neuropathy was perceived to be a barrier to healthy physical activity seems troublesome, in the light of the new scientific evidence regarding positive effects of physical activity on chemotherapy-induced neuropathy.^8^ The respondents were very aware of general health benefits of physical activity. However, due to their neuropathy, they felt too impaired or unmotivated to practice the recommended^8^ amount and intensity of physical activity. The respondents practiced integrative self-managing strategies, individualized to fit their own symptom experience, for example massage and distraction. More research is needed to evaluate these strategies’ efficacy, due to lack of scientific evidence for their efficacy on neuropathy so far.^8^ The current interviews gave a deeper understanding regarding the feasibility of the acupuncture procedure in terms of on how the individual patients experienced the therapeutic procedure. The independent interviewer made the interviews 3 months after the end of acupuncture instead of directly after the treatment period to prevent the patients from delivering interview data affected by a potential wish to “please the kind acupuncture-delivering therapist.” The interviews were aimed to give a deeper understanding of the patients’ situation and thus we included both genuine and sham acupuncture treated patients in the qualitative content analysis. One sham acupuncture treated patient claimed that the thumbs had become stiff during the treatment sessions. During the interviews none of the genuine acupuncture treated patients presented experiences of worsened symptoms—they described that the acupuncture was pleasant and valuable. This was important to secure since neuropathy symptoms worsened in a previous electro-acupuncture study.^22^ Knowledge regarding hyperalgesia associated with neuropathy in general^25^ raised concerns preceding this study regarding if acupuncture would be perceived as being pleasant or not. Rather surprisingly, but however in line with another study,^26^ our respondents also mentioned non-specific effects of acupuncture. They claimed that they were not sure if it was the acupuncture per se or the natural history and the entire therapeutic situation that had induced their improvements. Interestingly, these aspects are in line with previous literature presenting large impact of contextual effects for health outcomes,^21^ including during acupuncture. The patients expressed that they expected the acupuncture to be effective. They really had wished for effects since their symptom burden was high. Patients who did not notice any effects of the acupuncture therapy expressed that they believed acupuncture to be effective for other patients, often based on previous positive experiences of acupuncture. In line with the patients’ experiences, a prediction is based on learning from previous experience. Treatment expectations did highly modify treatment outcomes of previous interventions for pain in general.^46^ Patients experiencing chronic pain in general described that their benefit of the acupuncture therapy varied according to the “dosage” of the acupuncture received, and their relationship with the practitioner.^47^ These interviewed patients,^47^ like our respondents, described complete or partial relief of pain and disability.

Although acupuncture is a therapy commonly requested and offered at oncology departments^3,13^ as a part of integrative cancer care,^2^ it seems difficult to compare our study findings to other studies. As far as we know, there do not seem to be other sham-controlled studies published regarding manual acupuncture for persisting chemotherapy-induced neuropathy.^8^ Previous non-sham controlled studies indicate positive changes in neuropathy symptoms when undergoing acupuncture therapy.^14-17^ However, these studies did not control for non-specific treatment effects,^18-21,46^ sometimes cited as placebo effects. Similar to our study, Greenlee et al and co-workers^22^ designed their study to be able to control for non-specific treatment effects. Patients receiving electro-acupuncture and patients receiving sham electro-acupuncture during their chemotherapy-period reported a similar expected worsening in neuropathic pain during 3 months of neurotoxic chemotherapy. However, after the 4 months, the sham electro-acupuncture group had returned to baseline pain levels, while the pain-levels in the genuine electro-acupuncture group had continued to worsen.^22^ In that study, the patients were still undergoing chemotherapy. During ongoing chemotherapy, it does not seem to be suitable to apply penetrating acupuncture in the hands and feet, since needle penetration increases the peripheral blood flow.^24^ The microcirculation spreads the neurotoxic agents into the peripheral nerve endings, which induces nerve damages.^7^ Non-nociceptive sensorial stimulus often becomes painful during neuropathy.^25^ Our study indicates that the damaged peripheral nerve system did not perceive the sensorial stimulation of penetrating or non-penetrating acupuncture to be painful. Five of six patients in both the genuine and the sham acupuncture group considered the needling to be not or just mildly painful. However, the patients after the genuine acupuncture sessions unfurtunately experienced less reduction of unpleasant sensations in the hands compared to after the sham acupuncture sessions. The treated LI4 is a strong acupuncture point and it seems reasonable that acupuncture therapists may avoid discomfort by manipulating the LI4 point in a more gentle way, by choosing less strong acupuncture points, or avoiding needle penetration in the hands.

Two thirds of the patients receiving genuine or sham acupuncture experienced improved self-rated health after the 5-week treatment period, compared to before treatment. This was in line with previous studies presenting a strong relation between severity of neuropathy symptoms and level of impairments in capacity and quality of life.^5,6,11,12^ The genuine acupuncture group improveda median of 10 steps and the sham acupuncture group improved a median of 2 steps. The treated number of patients was small in our study and changes may sometimes occur just by chance; we did not perform any statistical group comparison of quality of life due to lack of statistical power. Accordingly, we still do not know if patients receiving genuine acupuncture would experience greater improvements in expereinced quality of life compared to sham acupuncture, highlighting the need for adding quality of life outcomes in future acupuncture studies.

The quality of our acupuncture pilot study may be reviewed using the revised Standards for Reporting Interventions in Clinical Trials of Acupuncture (STRICTA).^48^ STRICTA consists of 6 items, covering giving a rationale for the acupuncture, details of needling, the study setting and target population (e.g., the indication for the acupuncture), co-interventions, the practitioner’s background, and control intervention. Our study presented a solid rationale for the acupuncture. The methodology description presented all expected details on the needling. We did thoroughly describe the setting and target population, covering previously chemotherapy treated patients, and registered potential co-interventions. Since still recently conducted acupuncture studies were made without a credible sham-control,^15-17^ we belief that a strength is the randomized sham-controlled design, with blinded patients and evaluator. We used the credible telescopic sham device,^33^ resulting in satisfactorily blinded patients. Methodology literature on pilot studies^28^ encourages inclusion of a control group also in pilot studies, allowing for a realistic examination of implementation of blinded interventions and evaluation procedures. No attempts to blind the acupuncture-delivering therapist were made. Sham needles that may blind the therapist exist.^19^ However, since the therapist placed the Park et al ’s telescopic blunt sham device^33^ to sham points to avoid all sensorial stimulation of the traditional acupuncture points, the therapist could not be blinded. Further, the therapist obviously noticed that “deqi” occurred only during needle manipulation in the genuine acupuncture group.^32^ Telescopic sham needles are not entirely inert since they provide a low dose of sensorial stimulation on the treated sham-points^19^ that may induce social learned wellbeing effects.^18^ However, the only times the telescopic sham needles were pressed against the skin were when placing and manipulating the needles (totally about only 90 seconds per 5 weeks long treatment period), which is a very low dose of sensorial stimulation when discussing the role of touch for the mechanisms of action during acupuncture.^18^ Using the telescopic sham needles, we succeed to avoid the suggested effective specific ingredients of acupuncture, skin penetration of traditional acupuncture points and manipulation to the specific needle-sensation “deqi”, in the sham acupuncture group.^32^ Further, patients’ compliance with the treatment and data collection procedure was high.

The application of mixed methods was found to be beneficial; both quantitative and qualitative data were combined to provide a deeper understanding of patient experiences.^27^ The qualitative interview data^35^ was rich; so-called saturation^39^ occurred in terms of that the last interview covered no new experiences. Natural for being a pilot study,^28^ the study included few patients. The number of quantitatively observed treatment sessions was 120, in just 12 individual patients. We primarily studied several aspects of the feasibility of the study procedures,^28^ also covering the patients’ experiences. Secondarily to these feasibility aspects, we studied a scientific aspect,^28^ covering the pragmatically conducted evaluation of short-term effects of genuine acupuncture on the chemotherapy-induced neuropathic pain and unpleasant sensations. We are highly aware of that the evaluation did not comply with criteria for satisfactory statistical power.^28,40^

When randomly allocating high number of patients to treatment groups, different descriptive patient characteristics will distribute equally between the groups. This was not the case in our study; symptom-modifying descriptive patient characteristics,^9^ for example, gender and consumption of medications for other diseases, were skew distributed between the treatment groups. In line with recommendations regarding pilot studies,^28^ we did not present any statistical significance testing when descriptively comparing the randomization groups on pain, unpleasant sensations, and health regarding the entire treatment period, due to lack of statistical power when observing only 12 patients. When calculating the short-term effects, we calculated the changes between pre and post treatment VAS grading. Then we adequately provided *P*-values for the comparison between the genuine and sham acupuncture *sessions* (n = 60 genuine acupuncture treatment *sessions* compared to n = 60 sham acupuncture treatment *sessions*), rather than comparing the 12 individual patients (n = 6 genuine and n = 6 sham acupuncture treated patients). We chose to calculate descriptive statistics (n, %) and just simple comparative statistics, instead of more sophisticated analyze methods, due to low numbers of participants and not normally distributed data, as previously recommended.^28,40^ The data collection was conducted independently from the treating therapist, to minimize the impact of the therapist per se. When the patients delivered VAS data before or after each treatment session, they were not allowed to see their previously delivered VAS grading, to minimize expectancy effects.^46^ We used valid and reliable methods for data collection,^29,30,34,37,38^ and relevant statistical methods for analyzing VAS data,^40,41^ and conducted expectancy measuring^34^ to be able to control for a potential skew distribution of treatment expectations between the allocation groups. A limitation is still the limited generalizability; the study was small, and our observations are only valid for short-term effects since we did not analyze the long-term effects of acupuncture.

This pilot study indicates that patients experienced that the neuropathy negatively changed their life and that acupuncture was pleasant and valuable, but some patients presented doubts regarding its effect mechanisms. Patients receiving genuine acupuncture experienced short-term effects regarding pain and unpleasant sensations in the face compared to patients receiving sham acupuncture, while feet and hands did not improve. The patients were successfully blinded and complied with the acupuncture. We recommend cancer care practitioners to avoid strong needle stimulation in the hands and welcome future full-scaled randomized sham-controlled acupuncture studies evaluating long-term effects and cost-effectiveness of acupuncture for chemotherapy-induced neuropathy.
